# The Intestinal Fate of Citrus Flavanones and Their Effects on Gastrointestinal Health

**DOI:** 10.3390/nu11071464

**Published:** 2019-06-27

**Authors:** Yala Stevens, Evelien Van Rymenant, Charlotte Grootaert, John Van Camp, Sam Possemiers, Adrian Masclee, Daisy Jonkers

**Affiliations:** 1 Department of Internal Medicine, Division of Gastroenterology-Hepatology, School of Nutrition and Translational Research in Metabolism (NUTRIM), Maastricht University, P.O. Box 616, 6200 MD Maastricht, The Netherlands; 2 BioActor BV, Gaetano Martinolaan 85, 6229 GS Maastricht, The Netherlands; 3 Department of Food Technology, Safety and Health, Research Group Food Chemistry and Human Nutrition, Ghent University, Coupure Links 653, 9000 Ghent, Belgium; 4 ProDigest BVBA, Technologiepark 82, 9052 Ghent, Belgium

**Keywords:** citrus flavanones, intestinal microbiota, intestinal barrier function, intestinal inflammation, gastrointestinal health

## Abstract

Citrus flavanones, with hesperidin and naringin as the most abundant representatives, have various beneficial effects, including anti-oxidative and anti-inflammatory activities. Evidence also indicates that they may impact the intestinal microbiome and are metabolized by the microbiota as well, thereby affecting their bioavailability. In this review, we provide an overview on the current evidence on the intestinal fate of hesperidin and naringin, their interaction with the gut microbiota, and their effects on intestinal barrier function and intestinal inflammation. These topics will be discussed as they may contribute to gastrointestinal health in various diseases. Evidence shows that hesperidin and naringin are metabolized by intestinal bacteria, mainly in the (proximal) colon, resulting in the formation of their aglycones hesperetin and naringenin and various smaller phenolics. Studies have also shown that citrus flavanones and their metabolites are able to influence the microbiota composition and activity and exert beneficial effects on intestinal barrier function and gastrointestinal inflammation. Although the exact underlying mechanisms of action are not completely clear and more research in human subjects is needed, evidence so far suggests that citrus flavanones as well as their metabolites have the potential to contribute to improved gastrointestinal function and health.

## 1. Introduction

Polyphenols are naturally occurring secondary metabolites found in plants, where they play an important role in the plant’s defense systems by providing protection against, e.g., pathogens, insects, and UV radiation. Different classes of polyphenols can be identified, of which flavonoids are the largest and most studied group. Flavonoids have a widespread presence in edible plants and are a prominent part of the human diet. They can be classified based on their chemical structure into a variety of subclasses, such as flavanones, flavones, isoflavones, flavans (flavanols), anthocyanins, and flavonols. In Europe at the population level, a median daily flavonoid intake of 335.0 and 332.2 mg was found in men and women, respectively, of which about 5% could be attributed to flavanone consumption [1]. In contrast to other flavonoids that are present in a wide range of foods such as fruits, vegetables, cereals, legumes, and beverages like tea and red wine, the presence of flavanones in the human diet is mainly restricted to citrus fruits, and to a lower extent to tomatoes and aromatic herbs like mint [2]. Despite the fact that their presence is limited, flavanones contribute significantly to the dietary flavonoid intake because of the widespread consumption of citrus fruits and juices [3,4]. The main flavanone glycosides present in citrus fruits are hesperidin and naringin. Hesperidin is the principal flavanone in sweet oranges, while naringin is the most abundant flavanone in grapefruit and is primarily responsible for its distinct bitter taste [5]. Commercial sweet orange juice contains between 4.5 and 76.3 mg/100 mL hesperidin with an average of 37.5 mg/100 mL, whereas naringin concentrations in commercial grapefruit juice range between 4.8 and 119.7 mg/100 mL with an average of 43.5 mg/100 mL [6]. Overall, the average daily population intake of flavanones in Europe was found to be 25.7 ± 27.1 mg, being most often consumed as fruits (72.0%), juices (17.2%), wine (5.4%), and soft drinks (1.7%) [7].

The consumption of citrus flavanones has repeatedly been associated with a lower risk of degenerative diseases such as cardiovascular diseases and cancers [8,9,10,11,12,13]. This potentially protective effect has been related to the various properties of these compounds, which include anti-oxidative and anti-inflammatory activities [14,15,16]. Additionally, recent evidence has indicated that citrus flavanones could modulate the microbiota composition and activity by inhibiting pathogenic bacteria and selectively stimulating the growth of beneficial bacteria [17,18]. As inflammation, oxidative stress, and intestinal microbiota perturbations are involved in several gastrointestinal (GI) as well as metabolic diseases, consumption of citrus flavanones may contribute to the maintenance of intestinal homeostasis and may improve GI health. The intestinal microbiota has many functions that are important for host health. These include protection against pathogen colonization, maintenance of intestinal barrier function, for example, by secretion of mucus and regulation of the junctional complex, interaction with the host immune system, and its large metabolic capacity [19]. During normal intestinal homeostasis, the balance between inhibiting invading pathogens and tolerance to commensal microbes is tightly regulated [20,21]. However, a disturbed barrier function and/or reduced tolerance, may facilitate intestinal inflammation [22,23]. As part of the inflammatory response, reactive oxygen species and reactive nitrogen species are produced, resulting in enhanced exposure to oxidative stress [24].

Because of their putative mechanistic effects, citrus flavanones may be of interest in the prevention and/or treatment of various diseases. Most studies use extracts or purified bioactive compounds to study the effects of citrus flavanones. Their effects however, may be influenced by processes taking place in the GI tract such as bacterial metabolism and absorption. Therefore, in this review, we will provide an overview of the current evidence on the intestinal fate of citrus flavanones, including their interaction with the intestinal microbiota as well as their effects on factors contributing to GI health.

## 2. Intestinal Fate and Bioavailability

**Figure 1:**
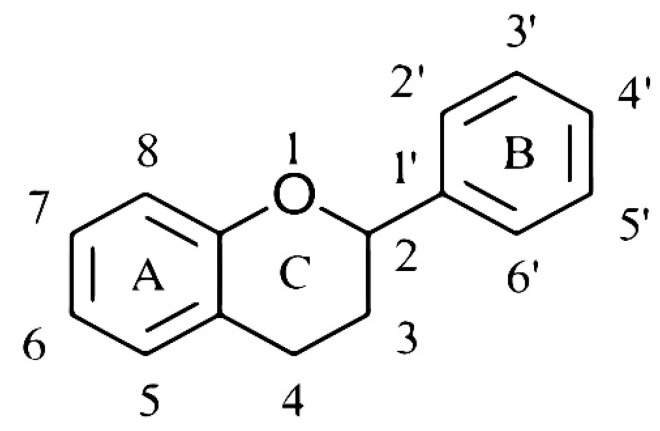
Basic chemical structure of flavonoids.

**Figure 2:**
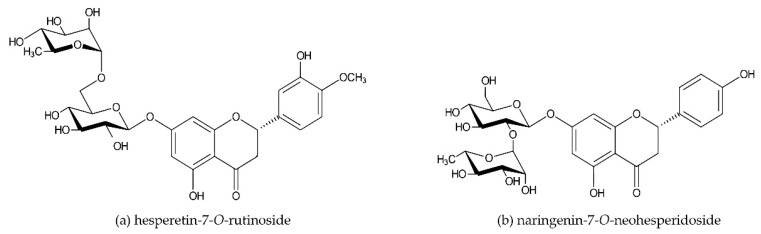
Chemical structure of hesperidin (**a**) and naringin (**b**).

The basic chemical structure of flavonoids consists of 15 carbon atoms, two aromatic rings (A and B), and a pyran ring (C) (Figure 1). Hesperidin and naringin are both flavanone glycosides. Their aglycones hesperetin and naringenin are attached to disaccharides consisting of glucose and rhamnose at the seventh-carbon position, with rhamnose as the terminal sugar (Figure 2a,b). Beneficial effects of citrus flavanones (e.g., radical scavenging and anti-inflammatory activity and modulation of microbiota) are considered to be related to their biochemical structure, such as the number and specific position of hydroxyl groups on the A and B ring and the presence of the sugar moiety [16,25,26,27].

The ability of citrus flavanones to exert beneficial effects strongly depends on their bioavailability, which can be affected by the structure of the compound, the food matrix, and host factors [28]. The intestinal metabolism of citrus flavanones is mainly determined by their degree of conjugation to sugar moieties [29,30,31] and the removal of these by intestinal bacteria [29]. Citrus flavanones such as hesperidin and naringin are considered to be largely resistant to enzymatic breakdown in the stomach and small intestine and, thereby, mainly reach the colon intact. Here, hesperidin and naringin are exposed to α-rhamnosidases secreted by the gut microbiota, which remove the rhamnose moiety followed by the removal of glucose by β-glucosidases [31,32,33]. Although the majority is converted in the colon, some breakdown can already take place in the distal part of the small intestine [34]. Upon release, the aglycones hesperetin and naringenin are absorbed through the intestinal epithelium by means of passive diffusion and proton-coupled active transport, or are further metabolized into phenolic acids and simple phenolics by C-ring cleavage, demethylation and dehydroxylation by bacterial enzymes [31,35,36,37,38]. Hence, the bioavailable fraction in the intestine upon citrus flavanone consumption is likely to consist of a mix of hesperetin, naringenin, and phenolic metabolites.

### 2.1. Intestinal Metabolism: In Vitro Evidence

Because of difficulties in sampling the small intestine and proximal colon, there are only limited data available on the biotransformation of citrus flavanones in the GI tract in vivo. Data from studies measuring fecal excretion levels show that these compounds have been metabolized extensively within the intestine, resulting in the formation of the aglycone forms and smaller phenolics [35,39]. Data from in vitro studies using various GI digestion models have provided additional information. In vitro digestion of hesperidin, naringin, and their aglycones has been carried out either in batch-like settings using incubations with fecal slurries or isolated bacteria [37,40,41], or in more comprehensive models, such as the TNO in vitro model of the colon (TIM-2) and the simulator of the human intestinal microbial ecosystem (SHIME) [42,43]. These in vitro studies, listed in Table 1, show quite consistently that during colonic microbial fermentation, hesperidin and naringin are first metabolized into their aglycones hesperetin and naringenin and subsequently into various phenolics, including dihydrocaffeic acid, isoferulic acid, 4-hydroxyphenylacetic acid, dihydroferulic acid, ferulic acid, resorcinol, phloroglucinol, 2,4-dihydroxyphenylacetic acid, 4-hydroxybenzoic acid, phloretic acid, phloroglucinic acid, hydrocinnamic acid, 3-(3′-hydroxyphenyl)propionic acid, protocatechuic acid, and hippuric acid. More information about the metabolism of citrus flavanones, including an overview of the proposed pathways, can also be found in a review by Kay et al. [44].

In addition to the type of metabolites formed, a recent study by Van Rymenant et al. provided information about the location of citrus flavanone metabolism. Using a digestion model comprising compartments representing the ascending, transverse, and descending colon (SHIME) inoculated with a fecal sample from a healthy volunteer, the metabolites formed upon hesperidin conversion were found at the highest concentrations in the compartments representing the ascending and transverse colon, while lower concentrations were measured in the descending colon [42]. These in vitro results suggest that microbial metabolism predominantly takes place in the more proximal parts of the colon. It should be noted that in vivo absorption mechanisms are lacking in these models. Furthermore, several of these studies used single or pooled samples for inoculation. Chen et al., investigating the in vitro biotransformation of naringin by intestinal bacteria from healthy human volunteers using labeled naringin, found that (intermediate) metabolites differed between subjects [45].

### 2.2. Bioavailability in Humans

Human studies performing analyses in plasma, urine, and/or feces also showed the formation of their aglycone forms as well as many smaller phenolic metabolites after the consumption of citrus flavanones (see also Table 2) [46,47,48,49,50]. These data indicate that citrus flavanones, like many other polyphenols, undergo extensive metabolism in vivo by the intestinal microbiota. Several of the metabolites identified in the in vitro simulations mentioned previously, were also found in these in vivo samples, suggesting that in vitro simulations have the capacity to mimic in vivo metabolism. It should be noted however that variations in flavanone metabolism can be found between individual subjects or donors both in vitro and in vivo, indicating that more research is needed. In addition, as most of these studies were performed in healthy volunteers or with fecal donations of healthy volunteers, it is unclear how disease states, dietary factors, and medication use may impact these findings. Although data are scarce, a few studies investigated the effects of the food matrix or medication use on the bioavailability of citrus flavanones. For example, the effects of the solubility of flavanones in orange juice and the ingestion of yoghurt together with orange juice on excretion levels of metabolites have been reported [46,51].

## 3. Effects on Microbiota Composition

The intestinal microbiota is a complex ecosystem which varies between individuals [52]. The interaction between the gut microbiota and polyphenols is considered to be bidirectional—in addition to the ability of intestinal bacteria to metabolize polyphenols, evidence has also accumulated that polyphenols may induce changes in the microbiota towards a more favorable composition and activity, including the production of short-chain fatty acids (SCFAs) in the colon. These metabolites have many known beneficial biological effects, e.g., acting as fuel for enterocytes, improving barrier function, and inhibiting inflammation [53,54]. Studies investigating the effect of citrus flavanones or food products derived from citrus fruit on the intestinal or fecal microbiota have mainly focused on their ability to inhibit the growth of pathogens, to increase beneficial commensal bacteria (such as *Bifidobacterium* and *Lactobacillus* species), and to stimulate the production of SCFAs.

### 3.1. In Vitro Studies

Duda-Chodak showed in vitro that both citrus flavanone aglycones, hesperetin and naringenin, inhibited the growth of different bacterial species after 24 h of incubation, while the parent compounds did not have such an effect. These included effects on *Bacteroides galacturonicus*, *Enterococcus caccae*, *Bifidobacterium catenulatum*, *Ruminococcus gauvreauii*, and *Escherichia coli*. The growth of *Lactobacillus* spp. was inhibited by naringenin only. Inhibitory effects were observed at concentrations of at least 250 μg/mL [18]. The ability of naringenin to inhibit bacterial growth was also confirmed by Parkar et al., who tested the effects on *E. coli*, *Staphylococcus aureus*, *Salmonella typhimurium,* and *Lactobacillus rhamnosus*. In this study, even lower minimum inhibitory concentrations were reported, being 62.5 μg/mL for *S. aureus* and 125 μg/mL for the other three strains [55]. Antibacterial activities of citrus flavanones have also been shown against vancomycin-intermediate *S. aureus* (VISA) at a concentration of 400 μg/mL for naringenin and 3200 μg/mL for naringin and hesperetin [56], and against *Aeromonas hydrophila* after 3125 μg/mL hesperidin treatment [57]. However, other studies failed to show the antibacterial activity of naringin, naringenin, or hesperidin against pathogens such as *S. aureus* and *E. coli* [58,59], but also found that hesperidin and naringin stimulated the growth of *Bifidobacterium bifidum* [60]. In addition to citrus flavanones and citrus flavanone aglycones, phenolics that might be formed as a result of colonic microbial fermentation can also influence the intestinal microbiota [55,60,61,62,63]. For example, antimicrobial effects towards *E. coli* have been reported for ferulic acid, isoferulic acid, 4-hydroxyphenylacetic acid, 4-hydroxybenzoic acid, vanillic acid, and caffeic acid, at concentrations ranging from approximately 100–1000 µg/mL depending on the specific phenolic acids and strains tested [55,61,62]. Interestingly, the previously mentioned study by Parkar et al. also tested the antimicrobial effect of caffeic acid and showed that naringenin was more effective at inhibiting the growth of the four different strains than caffeic acid [55]. On the other hand, Gwiazdowska et al. showed that incubation with caffeic acid and vanillic acid resulted in a stronger growth stimulation of *B. bifidum* than incubation with naringin and hesperidin [60].

The differences in the outcomes of the abovementioned studies may in part be due to differences in the experimental setup of these studies, such as the flavanone concentrations used. In vivo, it is possible that not the citrus flavanones themselves, but the metabolites formed as a result of microbial metabolism and cross-feeding are responsible for modulating the intestinal microbiota. Therefore, the results from studies that did not focus on single strains, but a more complete microbiota, may provide more insight into the effects that could be expected in the human GI tract.

In a study using the SHIME model, supplementation with 105 mL orange juice twice daily for 14 days resulted in a significant increase in *Lactobacillus* spp., *Bifidobacterium* spp., *Enterococcus* spp., and *Clostridium* spp. populations, while a decrease in enterobacteria was found. These changes in microbiota composition were accompanied by increased levels of the SCFAs acetate, butyrate, and propionate. Unfortunately, the concentration of citrus flavanones in the orange juice was not reported [17]. Changes in SCFA levels and microbiota composition were also reported in a recent study with a similar experimental setup in the SHIME model. After a three-week treatment period with 500 mg citrus extract, containing >80% hesperidin-2S and >4% of naringin, butyrate, and total SCFAs levels, and the relative abundance of the *Clostridium coccoides/Eubacterium rectale* cluster were significantly increased [42]. This cluster, which is part of *Clostridium* cluster XIVa, includes species that are known to produce butyrate [64]. Positive effects on SCFA levels have also been shown for pure naringin at concentrations of 40 and 160 mg/L, after 24 h of incubation in a batch-culture fermentation experiment [65].

### 3.2. Animal and Human Studies

In rats, three-week hesperetin supplementation via the diet (16.4 mmol/kg) resulted in significant increases in acetate and butyrate in the cecum. No significant effects were observed after hesperidin treatment. Similarly, hesperidin was not able to significantly affect the microbiota composition, while hesperetin administration resulted in increased proportions of *Clostridium* clusters IV and XVIII and a reduced proportion of *Clostridium* subcluster XIVa in the feces [66]. The differential effects are remarkable as hesperidin is expected to be rapidly converted into hesperetin in the proximal colon. However, in a recent rat study, oral administration of 100 and 200 mg/kg hesperidin three times a week for a period of four weeks, did result in significant changes in microbiota composition [67]. They found that hesperidin treatment, at the high dose, resulted in an increased *Lactobacillus* proportion, while an increased proportion of *Staphylococcus* and a decrease of *C. coccoides/E. rectale* were reported for both dosages.

The effect of hesperidin supplementation on microbiota composition and SCFAs has also been studied in humans. In a randomized, placebo-controlled trial in healthy subjects with features of metabolic syndrome, daily supplementation with 500 mg citrus extract (with >80% hesperidin-2S and >4% naringin) for 12 weeks, did result in an increase in the butyrate to total SCFA ratio but not in the absolute levels of fecal SCFAs [68].

In healthy volunteers, consumption of a pasteurized orange juice with unknown flavanone content for two months resulted in a significant increase in *Lactobacillus* spp. and total anaerobes in fecal samples. In addition, a significant reduction in ammonium concentration and an increase in the acetate to total SCFA ratio were found compared to the baseline [69]. Daily supplementation of two orange juices with different flavanone content for seven days in healthy volunteers resulted in microbiota composition shifts, of which the most notable was an increase in the abundance of Clostridia operational taxonomic units from Mogibacteriaceae, Tissierellaceae, Veillonellaceae, Odoribacteraceae, and Ruminococcaceae families [70].

Overall, results from available in vitro, animal and human studies show that citrus flavanone treatment can affect the composition of the microbiota or growth of specific taxa. Although some findings vary, growth inhibition of Enterobacteriaceae has been demonstrated repeatedly. Unfortunately, none of the human studies on the effect of flavanones on the microbiome included analyses on fecal metabolite levels.

## 4. Effects on Host Parameters Related to Gastrointestinal Health

According to Bischoff et al., GI health comprises an effective digestion and absorption, together with a stable microbiota, effective immune status, and state of general wellbeing in the absence of GI diseases [71]. In this context, an intact intestinal barrier is considered an important factor. One of its main functions is to act as defensive barrier against intraluminal toxins, antigens, and microorganisms. Together with the intestinal microbiota and the immune system, the barrier is determined by, e.g., the mucus layer and an intact epithelial cell monolayer sealed by junctional complexes [72]. Two in vitro studies using Caco-2 cell monolayers have shown that hesperetin and naringenin were able to improve intestinal barrier function, as measured by an increase in transepithelial electrical resistance and expression levels of tight junction proteins and a decrease in fluorescein isothiocyanate (FITC)-conjugated dextran flux across the monolayer [73,74]. Emerging evidence indicates that the intestinal permeability is increased in several disorders, such as inflammatory bowel disease (IBD) [75]. To study the effects of citrus flavanones on barrier function and other features of IBD, chemically induced colitis models in rodents have been widely used, such as dextran sulfate sodium (DSS)-, trinitrobenzene sulfonic acid (TNBS)-, and dinitrobenzene sulfonic acid (DNBS)-induced colitis. The key findings of in vitro and in vivo studies investigating the effects of citrus flavanones on host parameters related to intestinal inflammation and barrier function are summarized in Table 3. For example, animals treated with hesperidin, hesperetin, or naringenin showed a significant improvement of chemically induced colitis symptoms and inflammatory parameters, such as pro-inflammatory cytokines and neutrophil infiltration [76,77,78,79,80,81,82]. Beneficial effects were also shown with regard to colonic barrier function [76,77].

Furthermore, an improved barrier function and/or decrease in intestinal inflammation have also been reported in vitro for phenolics that can be formed during flavanone metabolism, such as ferulic acid, isoferulic acid, dihydroferulic acid, dihydrocaffeic acid, hydrocinnamic acid, and phloretic acid [83,84,85]. Studies directly comparing the effect size versus the flavanones or aglycones are not available.

At present, data from human studies regarding the effects of citrus flavanone supplementation in IBD or other GI diseases are lacking. However, in a recent human trial in healthy subjects with features of metabolic syndrome, supplementation with a citrus extract (consisting of >80% hesperidin-2S and >4% naringin) for 12 weeks showed a clear tendency to reduce levels of fecal calprotectin, a commonly used biomarker of intestinal inflammation [68]. Together with the abovementioned findings from in vitro and animal studies, this underlines the relevance to further investigate the effect of citrus flavanones in individuals with GI disorders characterized by intestinal inflammation, increased intestinal permeability, and/or microbial perturbations.

## 5. Conclusions

Following oral ingestion, citrus flavanones reach the distal part of the small intestine and the colon almost completely intact, where they interact with the microbiota. Evidence shows that citrus flavanones are extensively metabolized by intestinal bacteria resulting first in the formation of the aglycones hesperetin and naringenin and subsequently in the formation of various smaller phenolics. The microbiota composition and activity is highly variable between individuals, which can contribute to differences in metabolites formed and potential effects [30,86,87]. This can further be influenced by variations in dietary intake. Citrus flavanones and their metabolites, in turn, can also impact the microbiota composition and activity. For example, growth inhibition of Enterobacteriaceae has been reported. Beneficial effects of citrus flavanones on parameters such as GI inflammation and intestinal barrier function have repeatedly been reported, suggesting that intake of citrus flavanones can contribute to improved GI functioning and health. Based on the current evidence, this is likely a combined effect of the original compounds, their metabolites, and an interaction with the intestinal microbiome. Most of the currently available evidence is derived from in vitro and animal studies. Therefore, more research focusing on bioavailability and on effects in human subjects may help to improve our understanding of the effects of citrus flavanones in the human gut.

## Figures and Tables

**Table 1:** Nonexclusive listing of in vitro studies investigating the colonic metabolism of citrus flavanones.

In Vitro Model System [Ref.]	Sampling Time (Extra Sampling)	Treatment (Dosage)	Metabolites Formed	Comments
Batch incubation, fecal samples from four healthy volunteers [37]	24 h (1 h, 2 h, 4 h, 6 h, 8 h, 24 h)	Hesperetin (50 µmol in 50 mL) Naringenin (50 µmol in 50 mL)	Isoferulic acid, dihydrocaffeic acid, hydrocinnamic acid, 3-(3′-hydroxyphenyl)propionic acid, phloretic acid, 4-hydroxyphenylacetic acid Phloretic acid, hydrocinnamic acid, 4-hydroxyphenylacetic acid	Metabolite concentrations vary between time points and donors
Batch incubation, bacteria isolated from fresh human fecal samples [40]	12 h	Hesperidin (5 mg in 50 mL) Naringin (5 mg in 50 mL)	Hesperetin, resorcinol, phloroglucinol, 2,4-dihydroxyphenylacetic acid Naringenin, 4-hydroxybenzoic acid, phloroglucinol, phloroglucinic acid, 4-hydroxyphenylacetic acid	
Batch incubation, probiotic bacteria (*Bifidobacterium longum* R0175 and *Lactobacillus rhamnosus* subsp. *rhamnosus* NCTC 10302) [41]	48 h (12 h, 24 h, 36 h, 48 h)	Hesperidin (410 nmol in 10 mL) Naringenin (430 nmol in 10 mL) Hesperetin (820 nmol in 10 mL) Naringenin (920 nmol in 10 mL)	- - Isoferulic acid, dihydrocaffeic acid, 3-(3′-hydroxyphenyl)propionic acid, hydrocinnamic acid Phloretic acid, hydrocinnamic acid	Metabolite concentrations vary between time points and bacteria
SHIME, fecal sample from one healthy volunteer [42]	3 weeks (1 wk, 2 wks, 3 wks)	Hesperidin (500 mg)	Hesperetin, dihydrocaffeic acid, isoferulic acid, 4-hydroxyphenylacetic acid, dihydroferulic acid, ferulic acid, protocatechuic acid, vanillic acid, caffeic acid	
TIM-2, fecal samples from 10 healthy volunteers (pooled) [43]	28 h (0 h, 4 h, 8 h, 12 h, 16 h, 24 h, and 28 h)	Citrus + rutin supplement (284 mg rutin, 430 mg naringin, 88 mg hesperidin, 4.4 mg eriodictyol).	Phloretic acid, isoferulic acid, dihydroferulic acid, dihydrocaffeic acid, homovanillic acid, 3-hydroxyphenylacetic acid, 4-hydroxyphenylacetic acid, 2,4-dihydroxyphenylacetic acid, 3,4-dihydroxyphenylacetic acid, hippuric acid, resorcinol, phloroglucinol	
Batch incubation, fecal samples from 30 healthy volunteers [45]	24 h (4 h, 8 h, 12 h, 24 h)	[2′,3′,5′,6′-D4]naringin (10 µL (20 mg/mL) in 990 µL)	[2′,3′,5′,6′-D4]naringenin, 5-Oac-[2′,3′,5′,6′-D4]naringin, [2′,3′,5′,6′-D4]apiforol-7-O-rhamnoglucoside, 6/8-hydroxyl-[2′,3′,5′,6′-D4]naringin, 8/6-hydroxyl-[2′,3′,5′,6′-D4]naringin, [2′,5′,6′-D3]neoeriocitrin, [2′,3′,5′,6′-D4]apigenin, [2′,5′,6′-D3]eriodictyol, 6/8-hydroxyl-[2′,3′,5′,6′-D4]naringenin, 8/6-hydroxyl-[2′,3′,5′,6′-D4]naringenin, [2′,3′,5′,6′-D4]apiforol, 3-(4′-hydroxyphenyl)-[2′,3′,5′,6′-D4]propanoic acid, 3-phenyl-[2′,3′,5′,6′-D4]propanoic acid	Metabolism varies between donors

Various metabolites were found after in vitro colonic fermentation of citrus flavanones. A number of metabolites were formed in several different studies, but differences between studies have been observed, which might be due to differences in the methods and donors used. SHIIME, simulator of the human intestinal microbial ecosystem. TIM-2, TNO in vitro model of the colon.

**Table 2:** Nonexclusive listing of studies investigating the metabolism and bioavailability of citrus flavanones in human subjects.

Population (Design) [Ref.]	Treatment (Dosage/Flavanone Concentration)	Sample and Sampling Time (Extra Sampling)	Metabolites Formed	Comments
Healthy subjects, *n* = 7 (pre- and post-test) [46]	320 mg naringin	72 h urine (0–4 h, 4–8 h, 8–12 h, 12–24 h, 24–36 h, 36–48 h, 48–60 h, 60–72 h), 72 h feces	Urine and feces: 4-hydroxybenzoic acid, 4-hydroxyhippuric acid, hippuric acid, phoretic acid, phloretic acid sulfate, naringin, naringenin, naringenin diglucuronide Urine: naringenin diglucuronide, naringenin glucoside glucuronide, naringenin glucoside sulfate, naringenin glucuronide sulfate, naringin glucuronide, naringenin sulfate, hydroxylated naringenin glucuronide, naringenin glucuronide, naringenin glucuronide dimer, hydroxylated naringenin sulfate	Excretion of metabolites varied between individuals
Healthy volunteers, *n* = 12 (pre- and post-test) [47]	Orange juice (500 mL /398 μmol (poly)phenols, of which 246 μmol was hesperidin)	24 h plasma (0 h, 1 h, 2 h, 3 h, 4 h, 5 h, 6 h, 7 h, 8 h, 24 h)	Plasma: naringenin-4′-*O*-glucuronide, naringenin-7-*O*-glucuronide, naringenin-4′-sulfate, hesperetin-3′,7-*O*-diglucuronide, hesperetin-5,7-*O*-diglucuronide, hesperetin-3′,5-*O*-diglucuronide, hesperetin-*O*-glucuronyl-sulfate, hesperetin-7-*O*-glucuronide, hesperetin-3′-*O*-glucuronide, hesperetin-sulfate, hesperetin-3′-sulfate, eriodictyol sulfate, eriodictyol-*O*-glucuronyl-sulfate, caffeic acid-3′-sulfate, caffeic acid-4′-sulfate, ferulic acid, ferulic acid-4′-*O*-glucuronide, ferulic acid-4′-sulfate, isoferulic acid, isoferulic acid-3′-*O*-glucuronide, 3-(3′-hydroxyphenyl)hydracrylic acid, 3-(3′,4′-dihydroxyphenyl)propionic acid, 3-(3′-hydroxyphenyl)propionic acid-4′-sulfate, 3-(4′-hydroxyphenyl)propionic acid-3′-sulfate, 3-(3′-methoxy-4′-hydroxyphenyl)propionic acid, 3-(3′-methoxyphenyl)propionic acid-4′-*O*-glucuronide, 3-(3′-hydroxy-4′-methoxyphenyl)propionic acid, 3-(4′-methoxyphenyl)propionic acid-3′-*O*-glucuronide, 3-(3′-methoxyphenyl)propionic acid-4′-sulfate, 3-(4′-methoxyphenyl)propionic acid-3′-sulfate, 3-(4′-hydroxyphenyl)propionic acid, 3-(phenyl)propionic acid, hydroxyphenylacetic acid-*O*-glucuronide, hydroxyphenyl acetic acid-3′-sulfate, methoxyphenylacetic acid-*O*-glucuronide, 3′-methoxyphenylacetic acid-4′-sulfate, 4′-methoxyphenylacetic acid-3′-sulfate, 3′-hydroxyphenylacetic acid, 4′-hydroxyphenylacetic acid, 3,4-dihydroxybenzoic acid, benzoic acid-4-sulfate, 3′-methoxy-4′-hydroxymandelic acid, 4′-hydroxymandelic acid, hippuric acid-*O*-glucuronide, 3′-hydroxyhippuric acid, 4′-hydroxyhippuric acid, hippuric acid	
		24 h urine (baseline, 0–5 h, 5–8 h, 8–10 h, 10–24 h)	Urine: same as plasma, but also naringenin-4′,7-*O*-diglucuronide, naringenin-5,7-*O*-diglucuronide, naringenin-4′,5-*O*-diglucuronide, naringenin-*O*-glucuronyl-sulfate, hesperetin-5-*O*-glucuronide, hesperetin-*O*-glucosyl-sulfate, 3′-hydroxycinnamic acid, coumaric acid-3′-*O*-glucuronide, 4′-hydroxycinnamic acid, coumaric acid-4′-*O*-glucuronide, coumaric acid-4′-sulfate, caffeic acid-3′-*O*-glucuronide, caffeic acid-4′-*O*-glucuronide, 3-(3′-hydroxyphenyl)propionic acid-4′-*O*-glucuronide, 3-(phenyl)propionic acid-4′-*O*-glucuronide, 3-(4′-hydroxyphenyl)propionic acid-3-*O*-glucuronide, 3-(3′-hydroxyphenyl)propionic acid, 3-(phenyl)propionic acid-3′-*O*-glucuronide, 3-(phenyl)propionic acid-4′-sulfate,3-(phenyl)propionic acid-3′-sulfate, 3′,4′-dihydroxyphenylacetic acid, hydroxyphenylacetic acid-4′-sulfate, 3′-methoxy-4′-hydroxyphenylacetic acid, 3′,4′-dimethoxyphenylacetic acid, phenylacetic acid, hydroxybenzoic acid-*O*-glucuronide, 3-hydroxybenzoic acid-4-sulfate, 4-hydroxybenzoic acid-3-sulfate, 3-methoxy-4-hydroxybenzoic acid, 3-hydroxy-4-methoxybenzoic acid, 3-hydroxybenzoic acid, 4-hydroxybenzoic acid, benzoic acid-3-sulfate, 1,3,5-trihydroxyphenol, 1,2,3-trihydroxyphenol, 1,2-dihydroxyphenol	
Healthy subjects, *n* = 5 (controlled cross over) [48]	Orange juice (250 mL/168 µmol hesperidin, 12 µmol narirutin)	24 h urine (0–2 h, 2–5 h, 5–10 h, 10–24 h)	3-hydroxyphenylacetic acid, 3-hydroxyphenylhydracrylic acid, dihydroferulic acid, 3-methoxy-4-hydroxyphenylhydracrylic acid, 3-hydroxyhippuric acid	
Healthy volunteers, *n* = 12 (controlled cross over) [49]	Pulp-enriched orange juice (250 mL/537 µmol flavanones, of which 329 µmol was hesperidin)	24 h urine (0 h, 0–2 h, 2–5 h, 5–10 h, 10–24 h)	Hesperetin-*O*-diglucuronide, hesperetin-*O*-sulfate-*O*-glucuronide, hesperetin-*O*-sulfate-*O*-glucoside, hesperetin-7-*O*-glucuronide, hesperetin-3-*O*-glucuronide, hesperetin-3-*O*-sulfate, naringenin-*O*-diglucuronide, naringenin-4′-*O*-glucuronide, naringenin-7-*O*-glucuronide, eriodictyol-*O*-sulfate 3-(3′-hydroxy-4′-methoxyphenyl)hydracrylic acid, isoferulic acid, dihydroferulic acid, 3′-hydroxyhippuric acid, 4′-hydroxyhippuric acid, 3-(3′-hydroxyphenyl)hydracrylic acid, 3-methoxy-4-hydroxyphenylacetic acid, hippuric acid	Excretion of metabolites varied between individuals
Men at moderate CVD risk, *n* = 16 (controlled cross over) [50]	Orange juice, (767 mL/ 320 mg hesperidin)	5 h plasma	Hesperetin-glucuronide, naringenin-7-glucuronide, hesperetin-glucuronide, naringenin-glucuronide, hesperetin, naringenin Hippuric acid, dihydroferulic acid, dihydroferulic acid-3-glucuronide, 4-hydroxyphenylacetic acid, vanillic acid, hydroxyhippuric acid, iso/ferulic acid-glucuronide, 3- hydroxyhippuric acid, isovanillic acid, 3-hydroxyphenylacetic acid, vanillic acid-glucuronide, isovanillic acid-glucuronide, iso/vanillic acid-glucuronide, 4-hydroxy-benzoic acid, benzoic acid-4-glucuronide	

Various metabolites were found in plasma, urine, and/or fecal samples after ingestion of citrus flavanones in humans. Although a number of metabolites were formed in several studies, differences between studies have also been observed. These differences might be due to differences in study designs and host factors, including the microbiota. CVD, cardiovascular disease.

**Table 3:** Nonexclusive listing of studies on the effects of citrus flavanones on host parameters related to barrier function and inflammation.

Model [Ref.]	Treatment	Treatment Duration	Dose and Administration	Change in Main Outcomes vs. Control (Relevant Concentrations)
Caco-2 cell monolayers [73]	Hesperetin vs. control Naringenin vs. control	24 h	100 µM	**Barrier function**: ↑ TEER, occludin expression, claudin-4 expression, cytoskeletal association of occludin and claudin-1, and -3 ↔ FITC–dextran flux **Barrier function**: ↑ TEER, occludin expression, claudin-4 expression, cytoskeletal association of ZO-2, occludin and claudin-1, -3, and -4 ↔ FITC–dextran flux
Caco-2 cell monolayers [74]	Naringenin vs. control	48 h	10, 30, 100 µM	**Barrier function**: ↑ TEER (30, 100 µM)†, claudin-4 expression (30, 100 µM)†, cytoskeletal association of occludin, claudin-1, claudin-4 and ZO-2 (100 µM) ↓ FITC–dextran flux (30, 100 µM)†
DSS-induced colitis in male BALB/c mice [76]	Naringenin vs. control diet	9 days	0.3% of the diet, oral administration	**Barrier function**: ↓ Colonic permeability, claudin-1 expression ↑ Occludin, junctional adhesion molecule-A, claudin-3 expression **Inflammation**: ↓ DAI, colonic shortening, expression of cytokines (IFN-ϒ, IL-6, MIP-2, and IL-17A)
DSS-induced colitis in male BALB/c mice [77]	Naringenin vs. control diet Hesperetin vs. control diet	12 days	0.3% of the diet, oral administration 0.3% of the diet, oral administration	**Barrier function**: ↓ Colonic permeability ↑ Occludin expression **Inflammation**: ↓ Weight loss, colonic damage ↑ Colon length **Barrier function**: ↔ Colonic permeability, occludin expression **Inflammation**: ↓ Weight loss, colonic damage ↔ Colon length
DSS-induced colitis in male BALB/c mice [78]	Hesperidin vs. control	7 days	10, 40, 80 mg/kg, oral administration	**Inflammation**: ↓ DAI, MPO, MDA, IL-6, colonic wet weight (10, 40, 80 mg/kg)† ↓ Mucosal cell damage (80 mg/kg) ↔ IL-4 (10, 40, 80 mg/kg)
TNBS-induced colitis in female Wistar rats [79]	Hesperidin vs. control	Twice (48 h pre- + 48 h post-colitis induction)	2.5, 5, 10, 25, 50 mg/kg, oral administration	**Inflammation**: ↓ Colonic damage, colonic weight, colonic MPO (10, 25 mg/kg) ↑ Glutathione levels (10, 25 mg/kg), colonic fluid absorption (10–50 mg/kg) ↔ MDA, LTB4 (2.5–50 mg/kg)
TNBS- induced colitis in male Wistar rats [80]	Orange juice vs. control Grapefruit juice vs. control Combination vs. control	15 days	2 mL/kg, 5 mL/kg, 8 mL/kg, oral administration 0.1 mL/kg, 0.3 mL/kg, 0.5 mL/kg, oral administration 2 mL/kg OJ + 0.1 mL/kg GJ (low dose), 5 mL/kg OJ + 0.3 mL/kg GJ (high dose), oral administration	**Inflammation**: ↓ Colonic damage (2, 5, 8 mL/kg), MPO, CRP (5, 8 mL/kg), ALP (8 mg/kg) ↑ GSH (8 mL/kg) **Inflammation**:↓ Colonic damage (0.1, 0.3, 0.5 mL/kg) MPO, CRP (0.3, 0.5 mL/kg), ALP (0.3, 0.5 mL/kg) ↑ GSH (0.3 mL/kg) **Inflammation**: ↓ Colonic damage, MPO, CRP, ALP (low dose, high dose) ↑ GSH (high dose)
DNBS- induced colitis in Male CD1 mice [81]	Bergamot juice extract vs. control	4 days	5, 10, 20 mg/kg, oral administration	**Inflammation**: ↓ Colonic damage, weight loss, MPO, TNF-α, IL-1β, ICAM-1, p-selectin, nitrotyrosine, PAR, nuclear NF-kB translocation, p-JNK activation (20 mg/kg) ↑ Colon length (20 mg/kg)
LPS-challenged broiler chickens [82]	Hesperidin vs. control diet	42 days	20 mg/kg diet, oral administration	**Inflammation**: ↑ Phagocytic index, villus length, villus width, villus length/crypt depth ↓ Crypt depth ↔ Body weight gain, feed intake feed conversion ratio
Human subjects with features of metabolic syndrome [68]	Citrus extract (>80% hesperidin-2S and <4% of naringin) vs. placebo	12 weeks	500 mg, oral administration	**Inflammation**: ↔ Calprotectin

TEER, transepithelial electrical resistance. FITC, fluorescein isothiocyanate. ZO-2, *zonula occludens 2.* DSS, dextran sulfate sodium. DAI, disease activity index. IFN-ϒ, interferon-ϒ. IL, interleukin. MIP-2, macrophage inflammatory protein-2. MPO, myeloperoxidase. MDA, malondialdehyde. TNBS, trinitrobenzene sulfonic acid. LBT4, leukotriene B4. OJ, orange juice. GJ, grapefruit juice. CRP, C-reactive protein. ALP, *alkaline phosphatase.* GSH, glutathione. DNBS, dinitrobenzene sulfonic acid. TNF-α, tumor necrosis factor-α. ICAM-1, intercellular adhesion molecule-1. PAR, poly ADP-ribose. NF-kB, nuclear factor-kB. ↑, increase. ↓, decrease. ↔, no significant change. †, dose-dependent effect.
